# Novel Polymyxin Combination With Antineoplastic Mitotane Improved the Bacterial Killing Against Polymyxin-Resistant Multidrug-Resistant Gram-Negative Pathogens

**DOI:** 10.3389/fmicb.2018.00721

**Published:** 2018-04-12

**Authors:** Thien B. Tran, Jiping Wang, Yohei Doi, Tony Velkov, Phillip J. Bergen, Jian Li

**Affiliations:** ^1^Monash Biomedicine Discovery Institute, Department of Microbiology, School of Biomedical Sciences, Faculty of Medicine, Nursing and Health Sciences, Monash University, Melbourne, VIC, Australia; ^2^Drug Delivery, Disposition and Dynamics, Monash Institute of Pharmaceutical Sciences, Monash University, Melbourne, VIC, Australia; ^3^Division of Infectious Diseases, Department of Medicine, University of Pittsburgh Medical Center, Pittsburgh, PA, United States; ^4^Department of Pharmacology and Therapeutics, School of Biomedical Sciences, Faculty of Medicine, Dentistry and Health Sciences, The University of Melbourne, Melbourne, VIC, Australia; ^5^Centre for Medicine Use and Safety, Monash Institute of Pharmaceutical Sciences, Monash University, Melbourne, VIC, Australia

**Keywords:** polymyxin, mitotane, repurposing, combination therapy, multidrug-resistance

## Abstract

Due to limited new antibiotics, polymyxins are increasingly used to treat multidrug-resistant (MDR) Gram-negative bacteria, in particular carbapenem-resistant *Acinetobacter baumannii*, *Pseudomonas aeruginosa*, and *Klebsiella pneumoniae*. Unfortunately, polymyxin monotherapy has led to the emergence of resistance. Polymyxin combination therapy has been demonstrated to improve bacterial killing and prevent the emergence of resistance. From a preliminary screening of an FDA drug library, we identified antineoplastic mitotane as a potential candidate for combination therapy with polymyxin B against polymyxin-resistant Gram-negative bacteria. Here, we demonstrated that the combination of polymyxin B with mitotane enhances the *in vitro* antimicrobial activity of polymyxin B against 10 strains of *A. baumannii*, *P. aeruginosa*, and *K. pneumoniae*, including polymyxin-resistant MDR clinical isolates. Time-kill studies showed that the combination of polymyxin B (2 mg/L) and mitotane (4 mg/L) provided superior bacterial killing against all strains during the first 6 h of treatment, compared to monotherapies, and prevented regrowth and emergence of polymyxin resistance in the polymyxin-susceptible isolates. Electron microscopy imaging revealed that the combination potentially affected cell division in *A. baumannii*. The enhanced antimicrobial activity of the combination was confirmed in a mouse burn infection model against a polymyxin-resistant *A. baumannii* isolate. As mitotane is hydrophobic, it was very likely that the synergistic killing of the combination resulted from that polymyxin B permeabilized the outer membrane of the Gram-negative bacteria and allowed mitotane to enter bacterial cells and exert its antimicrobial effect. These results have important implications for repositioning non-antibiotic drugs for antimicrobial purposes, which may expedite the discovery of novel therapies to combat the rapid emergence of antibiotic resistance.

## Introduction

The emergence of Gram-negative bacteria with resistance to multiple classes of antibiotics is causing serious problems for health care centers worldwide (Boucher et al., 2013). Infections caused by multidrug-resistant (MDR) Gram-negative bacteria not only have higher mortality rates (Harris et al., 2015) but also lead to more economic burden than infections caused by susceptible Gram-negative bacteria (Gandra et al., 2014). Among these MDR Gram-negative bacteria, carbapenem-resistant *Acinetobacter baumannii* has been identified as one of the most difficult-to-treat pathogens and is becoming increasingly problematic for critically ill patients and war-wounded soldiers (Davis et al., 2005; Gupta et al., 2006; Peleg et al., 2008; Centers for Disease Control and Prevention [CDC], 2013). *A. baumannii* possesses numerous mechanisms of carbapenem resistance (Tiwari et al., 2012a,b; Tiwari and Moganty, 2014; Roy et al., 2017; Verma et al., 2017), and can cause a wide range of infections including pneumonia, urinary tract and wound infections, bacteremia, and meningitis (Gupta et al., 2006; Maragakis and Perl, 2008; Tiwari et al., 2012a). More recently, the World Health Organization (WHO) has classified carbapenem-resistant *A. baumannii*, *Pseudomonas aeruginosa*, and Enterobacteriaceae as the top priorities for research and development of new antibiotics (Tacconelli and Magrini, 2017).

Due to the current lack of effective antibiotics against MDR Gram-negative bacteria, the polymyxins (colistin and polymyxin B) have been revived as antibiotics of last resort (Li et al., 2006; Nation et al., 2015). However, resistance to polymyxins is on the rise (Marchaim et al., 2011; Kim et al., 2014; Goli et al., 2016) and a growing body of evidence suggests that resistance to polymyxins can emerge with monotherapy (Tam et al., 2005; Tan et al., 2007; Bergen et al., 2011; Meletis et al., 2011; Deris et al., 2012; Hermes et al., 2013; Lee et al., 2013; Ly et al., 2015; Lenhard et al., 2017; Zhao et al., 2017). *In vitro* studies have revealed that polymyxin resistance may occur within 24 h after colistin or polymyxin B monotherapy (Bergen et al., 2011; Deris et al., 2012; Tran et al., 2016). The two main mechanisms of polymyxin resistance identified in Gram-negative bacteria are lipid A modifications and loss of LPS (Moffatt et al., 2010; Arroyo et al., 2011).

Unfortunately, the *de novo* drug discovery and development process is lengthy (usually 10–17 years) and has a low success rate (<10%; Ashburn and Thor, 2004). With limited new antibiotics in the pipeline, an approach to expedite the discovery process is through the repositioning of non-antibiotic FDA-approved drugs. This process can be as short as 3 years as these drugs have already passed the FDA safety requirements and have well-defined pharmacokinetics (Ashburn and Thor, 2004). In light of the dire resistance problem, we screened an FDA drugs’ library to identify potential synergistic candidates with polymyxins for the treatment of MDR Gram-negative bacteria. Our screening identified FDA-approved antineoplactic mitotane as a highly potential non-antibiotic candidate for combination therapy with polymyxin B. In this study, we evaluated the *in vitro* antimicrobial activity of the combination of polymyxin B and mitotane against highly resistant clinical isolates of Gram-negative bacteria including carbapenem-resistant *A. baumannii*, carbapenem-resistant *P. aeruginosa*, and New Delhi metallo-β-lactamase (NDM)-producing *Klebsiella pneumoniae*. Our findings highlight the potential of this novel polymyxin/non-antibiotic combination for treatment of these problematic Gram-negative “superbugs”.

## Materials and Methods

### Bacterial Isolates

Ten bacterial strains which included multidrug- and polymyxin-resistant isolates were examined in this study (**Table 1**). *A. baumannii* ATCC 17978, *A. baumannii* ATCC 19606, *K. pneumoniae* ATCC 13883, and *P. aeruginosa* ATCC 27853 were obtained from the American Type Culture Collection (Rockville, MD, United States). *A. baumannii* FADDI-AB225 (formally designated ATCC 17978-R2) is a polymyxin-resistant *pmrB* mutant (due to phosphoethanolamine-modified lipid A) derived from ATCC 17978 (Arroyo et al., 2011). *A. baumannii* FADDI-AB065 (formally designated ATCC 19606R) is a polymyxin-resistant, LPS-deficient, *lpxA* mutant derived from ATCC 19606 (Moffatt et al., 2010). Polymyxin-susceptible *A. baumannii* FADDI-AB180 (formally designated 2949) and lipid A modified (with phosphoethanolamine and galactosamine) polymyxin-resistant *A. baumannii* FADDI-AB181 (formally designated 2949A) are carbapenem-resistant MDR clinical isolates from the bronchoalveolar lavage fluid of a patient before and after colistin therapy (Pelletier et al., 2013). *P. aeruginosa* FADDI-PA070 is a non-mucoid, MDR (including carbapenem- and polymyxin-resistant) clinical isolate from the sputum of a patient with cystic fibrosis (formally designated 19147 n/m; Bergen et al., 2011). *K. pneumoniae* FADDI-KP027 is a polymyxin-resistant, NDM-producing clinical isolate from the sputum of a patient with respiratory tract infection. Isolates were stored in tryptone soy broth (Oxoid) with 20% glycerol (Ajax Finechem, Seven Hills, NSW, Australia) in cryovials at -80°C and subcultured onto nutrient agar plates (Media Preparation Unit, University of Melbourne, Melbourne, VIC, Australia) before use.

**Table 1:** Minimum inhibitory concentrations (MICs) for polymyxin B and mitotane against bacterial isolates examined in this study.

Bacterial isolate (former nomenclature)	MIC (mg/L)			
	Polymyxin B	Mitotane	Mitotane in the presence of 2 mg/L polymyxin B	Polymyxin susceptibility and mechanism of resistance
*A. baumannii* ATCC 17978	0.25	>128	–	Susceptible
*A. baumannii* FADDI-AB225 (ATCC 17978-R2)^PR^	16	>128	4	Lipid A modification
*A. baumannii* ATCC 19606	0.5	>128	–	Susceptible
*A. baumannii* FADDI-AB065 (ATCC 19606R)^PR^	64	4	4	LPS loss
*A. baumannii* FADDI-AB180 (2949)^MDR^	1	>128	–	Susceptible
*A. baumannii* FADDI-AB181 (2949A)^MDR,PR^	64	>128	4	Lipid A modification
*P. aeruginosa* ATCC 27853	0.5	>128	–	Susceptible
*P. aeruginosa* FADDI-PA070 (19147 n/m)^MDR,PR^	64	>128	4	Uncharacterized
*K. pneumoniae* ATCC 13883	0.5	>128	–	Susceptible
*K. pneumoniae* FADDI-KP027^MDR,PR^	256	>128	4	Uncharacterized


*^*MDR*^, multidrug-resistant: defined as non-susceptible to ≥1 treating agent in ≥3 antimicrobial categories (Magiorakos et al., 2012). ^*PR*^, polymyxin resistant: defined as an MIC of ≥4 mg/L for *Acinetobacter* spp. and ≥8 mg/L for *P. aeruginosa* as per CLSI guideline (Clinical and Laboratory Standards Institute [CLSI], 2016); and >2 mg/L for Enterobacteriaceae as per EUCAST guidelines (The European Committee on Antimicrobial Susceptibility Testing [EUCAST], 2017); and mitotane breakpoints are not available. –, Not performed.*

### Antimicrobial Agents and Susceptibility Testing

Polymyxin B (Beta Pharma, China; batch number 20120204) solutions were prepared in Milli-Q water (Millipore, North Ryde, NSW, Australia) and sterilized using a 0.20-μm cellulose acetate syringe filter (Millipore, Bedford, MA, United States). Mitotane (Sigma-Aldrich, Australia; lot number BCBG9480V) solutions were prepared in dimethyl sulfoxide (DMSO; Sigma-Aldrich, Australia). Stock solutions were stored at -20°C for no longer than 1 month. The minimum inhibitory concentrations (MICs) to polymyxin B and mitotane were determined for all isolates in three replicates on separate days using broth microdilution with cation-adjusted Mueller–Hinton broth (CAMHB; Oxoid, England; 20–25 mg/L Ca^2+^ and 10–12.5 mg/L Mg^2+^) according to the Clinical and Laboratory Standards Institute guidelines (Clinical and Laboratory Standards Institute [CLSI], 2016). Stock solutions of polymyxin B were diluted to the desired concentrations in CAMHB, while mitotane was initially prepared in DMSO and subsequently in CAMHB to obtain the desired drug concentrations with 10% DMSO (v/v). The procedure to measure the MICs of polymyxin B and mitotane was adapted from our previous method (Tran et al., 2016). Briefly, 100 μL of the bacterial suspension (10^6^ cfu/mL) was combined with 100 μL of the prepared polymyxin B solutions or 50 μL of CAMHB plus 50 μL of the prepared mitotane solutions in 96-well microtiter plates (Techno Plas, St Marys, SA, Australia). For mitotane MICs, the final concentration of 2.5% DMSO (v/v) was employed, as preliminary studies demonstrated that 2.5% DMSO (v/v) had no effect on the bacterial growth. The plates were incubated standing at 37°C for 20 h and MICs were determined as the lowest drug concentrations that inhibited the visible growth of the bacteria. For polymyxin-resistant isolates, MICs of mitotane in the presence of 2 mg/L of polymyxin B were also determined. According to the CLSI guidelines, polymyxin B MIC is ≤2 mg/L for polymyxin-susceptible *A. baumannii* and *P. aeruginosa*, ≥4 mg/L for polymyxin-resistant *A. baumannii*, and ≥8 mg/L for polymyxin-resistant *P. aeruginosa* (Clinical and Laboratory Standards Institute [CLSI], 2016). For *K. pneumoniae*, breakpoints have not yet been established by the CLSI. Consequently, susceptibility to polymyxin B was extrapolated from the European Committee on Antimicrobial Susceptibility Testing (EUCAST) colistin breakpoints where susceptibility is defined as an MIC ≤2 mg/L and resistance an MIC of >2 mg/L (The European Committee on Antimicrobial Susceptibility Testing [EUCAST], 2017).

### Time-Kill Studies

Time-kill studies were conducted for all isolates based on our previously described method (Tran et al., 2016). Briefly, bacteria were grown overnight in 20 mL CAMHB. The overnight broth cultures were transferred to 20 mL of fresh CAMHB at ∼50–100-fold dilutions and incubated for an additional 3–4 h to generate log-phase culture at ∼0.55 McFarland standard. The log-phase cultures were transferred to 20 mL of fresh CAMHB at ∼100-fold dilution in borosilicate glass tubes for treatment to minimize loss of drug due to non-specific binding to the plastic. For the drug-containing tubes, polymyxin B, mitotane, or both compounds were added to achieve final concentrations of 2 mg/L for polymyxin B and 4 mg/L for mitotane (the minimum concentration of mitotane identified by broth microdilution assay to inhibit to growth of polymyxin-resistant isolates in the presence of 2 mg/L polymyxin B). The final concentration of 0.4% DMSO (v/v) was achieved for all treatments; 2.5% DMSO (v/v) had no effect on bacterial growth with 2 mg/L of polymyxin B (Tran et al., 2016). Samples (1 mL) were aseptically removed at 0, 0.5, 1, 2, 4, 6, and 24 h and inoculated onto nutrient agar plates for viable-cell counting. Colonies were counted after 24 h incubation at 37°C using a ProtoCOL colony counter (Synbiosis, Cambridge, United Kingdom). The combination of polymyxin B and mitotane was considered synergistic if the bacterial killing was ≥2 log_10_ compared to the most active monotherapy (Pillai et al., 2005). Changes to polymyxin B MICs were determined for all cultures that showed regrowth after 24 h to evaluate the emergence of polymyxin resistance.

### Phase Contrast, Scanning Electron, and Transmission Electron Microscopy

Phase contrast microscopy, scanning electron microscopy (SEM), and transmission electron microscopy (TEM) were employed to examine the effect of the polymyxin B/mitotane combination on the cellular morphology of polymyxin-susceptible *A. baumannii* ATCC 17978 and polymyxin-resistant *A. baumannii* FADDI-AB225. Bacteria were subcultured and treated with 2 mg/L polymyxin B, 4 mg/L mitotane, or both antibiotics for 2 h in CAMHB as per the time-kill studies. For phase contrast microscopy, 20 μL of each culture was used to prepare wet samples for instant observation on a phase contrast microscope. For the SEM and TEM studies, samples were transferred to 50-mL polypropylene tubes (Greiner Bio-One, Frickenhausen, Germany) and centrifuged at 3220 ×*g* for 10 min three times. Between centrifugation steps, supernatants were discarded and bacterial pellets resuspended and washed in 1 mL phosphate buffered saline (PBS). Following the final centrifugation step, the supernatants were removed and bacterial pellets resuspended and fixed in 0.5 mL 2.5% glutaraldehyde in PBS. The tubes were left in a rocker shaker for 20 min at room temperature. Once fixed, tubes were centrifuged at 3220 ×*g* for 10 min, the fixatives removed, and bacterial pellets washed twice in 1 mL PBS as above. Pellets were finally resuspended in 1 mL PBS, and SEM and TEM were conducted at the Department of Botany, University of Melbourne, Australia.

### Mouse Burn Wound Infection Model

A mouse burn wound infection model was employed to assess the *in vivo* antimicrobial activity of the polymyxin B/mitotane combination against polymyxin-resistant *A. baumannii* FADDI-AB225. Bacterial inoculums were prepared with early log-phase culture. After centrifugation at 3220 ×*g* for 10 min, the supernatant was removed and bacterial cell pellets were suspended in 0.9% saline to approximately 10^9^ cfu/mL. Bacterial samples (100 μL) were then loaded into 29-G 0.3-mL insulin syringes for inoculation of burn wounds. Drug solutions were prepared by initially dissolving mitotane in polyethylene glycol (PEG) 200 to ∼4,096 mg/L and polymyxin B in 0.9% saline to ∼1,536 mg/L. An equal amount of the two drug solutions was later combined to produce the combination solution with ∼2,048 mg/L mitotane and 768 mg/L polymyxin B. For mitotane monotherapy, mitotane solution was combined with an equal volume of 0.9% saline. For polymyxin B monotherapy, polymyxin B was combined with an equal volume of PEG 200. For solvent controls, equal volumes of blank PEG 200 and 0.9% saline were combined. Prior to infection, female NIH Swiss mice (6–10 weeks old, ∼30 g body weight) were sedated with isoflurane and anesthesia was maintained throughout the entire procedure. Hair from the mouse dorsal skin was removed and the local skin area was injected with 100 μL of Bupivacaine (Marcaine 0.5%). A burn wound was established with a hot iron bolt from boiling water and bacteria injected into the burn eschar. After 2 h, different treatments were applied topically by evenly spreading 200 μL of the drug solutions across the wounds of groups of four mice. This study included five groups of four mice comprising blank control (no treatment), solvent control, polymyxin B monotherapy, mitotane monotherapy, and the combination (polymyxin B and mitotane). Each wound of the treated groups received 154 μg of polymyxin B (0.5%, w/w), 410 μg of mitotane (1.4%, w/w), or both. Four hours after treatment, mice were sacrificed and the burn wound skin tissues and the muscle tissue (∼0.3 g) under the burn wounds were aseptically removed and placed separately into 8 mL of sterile saline in 50-mL Falcon tubes. Burn wound skin tissues were homogenized under sterile conditions and filtered using a filter bag (Bag Stomacher Filter Sterile, Pore Size 280 micrometer, 0.5 cm × 16 cm, Labtek Pty Ltd.). Filtrate (1 mL) was then transferred into a sterile test tube for serial dilution and 100 μL was cultured onto nutrient agar for viable counting. Viable counts were performed on the next day following overnight incubation at 37°C. Statistical significance for the bacterial killing of different treatment groups was calculated with one-way ANOVA and Tukey’s multiple comparisons (Tukey’s HSD).

## Results

### MICs of Polymyxin B and Mitotane Against Polymyxin-Susceptible and -Resistant Isolates of *A. baumannii*, *P. aeruginosa*, and *K. pneumoniae*

The polymyxin B and mitotane MICs against all 10 Gram-negative isolates are shown in **Table 1**. Additionally, **Table 1** shows the MICs of mitotane in the presence of 2 mg/L polymyxin B against the polymyxin-resistant isolates. Apart from *A. baumannii* FADDI-AB065, mitotane monotherapy had no antimicrobial activity at concentrations up to 128 mg/L. However, in the presence of 2 mg/L polymyxin B, 4 mg/L of mitotane was effective at inhibiting growth of five polymyxin-resistant isolates (**Table 1**).

The changes to the polymyxin B MICs of 10 examined isolates after overnight treatment with either polymyxin B monotherapy, mitotane monotherapy, or polymyxin B/mitotane combination are shown in **Table 2**. In the control group (overnight incubation in drug-free CAMHB), polymyxin B MICs of all isolates at 24 h were not affected as all values remained within two folds of the baseline MICs (European Committee for Antimicrobial Susceptibility Testing (EUCAST) of the European Society of Clinical Microbiology and Infectious Diseases (ESCMID), 2003). After treatment with polymyxin B monotherapy at 2 mg/L, polymyxin B MICs of the polymyxin-resistant isolates at 24 h remained unchanged. However, with the three polymyxin-susceptible isolates that showed regrowth at 24 h, polymyxin B MICs of the 24-h samples increased significantly (≥32 times). Following mitotane monotherapy at 4 mg/L, polymyxin B MICs remained unchanged for all polymyxin-susceptible isolates and three polymyxin-resistant isolates; the polymyxin B MIC of polymyxin-resistant *A. baumannii* FADDI-AB065 at 24 h could not be determined, as it was highly susceptible to mitotane and showed no regrowth after 24 h. Interestingly, the polymyxin B MIC of polymyxin-resistant *A. baumannii* FADDI-AB225 was reduced significantly (32-fold lower than the baseline) after 24-h exposure to mitotane. In the combination treatment group, the polymyxin B MICs did not change for all four polymyxin-resistant isolates that showed regrowth after 24 h.

**Table 2:** Changes in baseline polymyxin B MICs following overnight treatment with polymyxin B (PMB) monotherapy, mitotane (MIT) monotherapy, and polymyxin B/mitotane combination.

Bacterial isolate	Polymyxin B MICs relative to their baseline values			
	Control	PMB 2 mg/L	MIT 4 mg/L	PMB 2 mg/L + MIT 4 mg/L
*A. baumannii* ATCC 17978	2 × MIC	NG	2 × MIC	NG
*A. baumannii* FADDI-AB225	2 × MIC	2 × MIC	1/32 × MIC	1 × MIC
*A. baumannii* ATCC 19606	1 × MIC	32 × MIC	1 × MIC	NG
*A. baumannii* FADDI-AB065	1 × MIC	1 × MIC	NG	NG
*A. baumannii* FADDI-AB180	1 × MIC	32 × MIC	1 × MIC	NG
*A. baumannii* FADDI-AB181	1 × MIC	1 × MIC	2 × MIC	1 × MIC
*P. aeruginosa* ATCC 27853	2 × MIC	NG	1 × MIC	NG
*P. aeruginosa* FADDI-PA070	1 × MIC	1 × MIC	1 × MIC	1 × MIC
*K. pneumoniae* ATCC 13883	1/2 × MIC	64 × MIC	1/2 × MIC	NG
*K. pneumoniae* FADDI-KP027	1 × MIC	1 × MIC	1 × MIC	1 × MIC


*NG, no growth at 24 h.*

### Time-Kill Results for Polymyxin B and Mitotane Against Polymyxin-Susceptible and -Resistant Isolates of *A. baumannii*, *P. aeruginosa*, and *K. pneumoniae*

**FIGURE 1:**
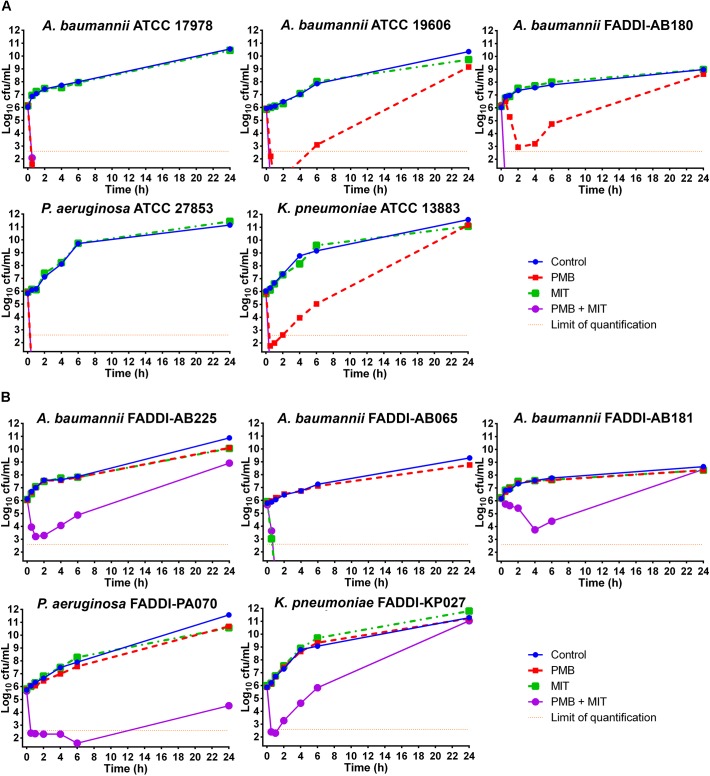
Time-kill kinetics of polymyxin B (PMB; 2 mg/L) and mitotane (MIT; 4 mg/L) monotherapy and combination therapy against five polymyxin-susceptible Gram-negative isolates **(A)** and five polymyxin-resistant Gram-negative isolates **(B)**. The *y*-axis starts from the limit of detection and the limit of quantification is indicated by the orange dotted line.

Time-kill profiles for polymyxin B and mitotane mono- and combination therapy are shown in **Figure 1**. Against the five polymyxin-susceptible isolates, polymyxin B monotherapy (2 mg/L) showed effective bacterial killing within 6 h with a minimum of ∼3 log_10_ cfu/mL killing (FADDI-AB180) and ∼6 log_10_ cfu/mL killing for the remaining susceptible isolates; however, regrowth to control values occurred by 24 h with three isolates (**Figure 1A**). There was no bacterial killing of polymyxin-susceptible isolates with mitotane monotherapy (4 mg/L), with growth comparable to that of controls (**Figure 1A**). With the combination, bacterial counts for all five polymyxin-susceptible isolates were reduced to below the limit of detection within 0.5–1 h, with no viable colonies detected thereafter (**Figure 1A**). Against the five polymyxin-resistant isolates, 2 mg/L polymyxin B monotherapy was ineffective with growth paralleling that of the controls (**Figure 1B**). Similarly, mitotane monotherapy displayed no antimicrobial activity against four of the five isolates (**Figure 1B**). However, against *A. baumannii* FADDI-AB065 mitotane monotherapy reduced bacterial counts to below the level of detection within the first 0.5 h and prevented regrowth over 24 h. Combination treatment showed synergistic bacterial killing (i.e., >2 log_10_ reduction compared to the most active monotherapy) between 0.5 and 6 h with the remaining four isolates; interestingly, regrowth occurred at 24 h in all four cases and was close to control values in three cases (**Figure 1B**).

### Impact of Polymyxin B and Mitotane Treatment on the Cellular Morphology of Polymyxin-Susceptible and -Resistant *A. baumannii*

**FIGURE 2:**
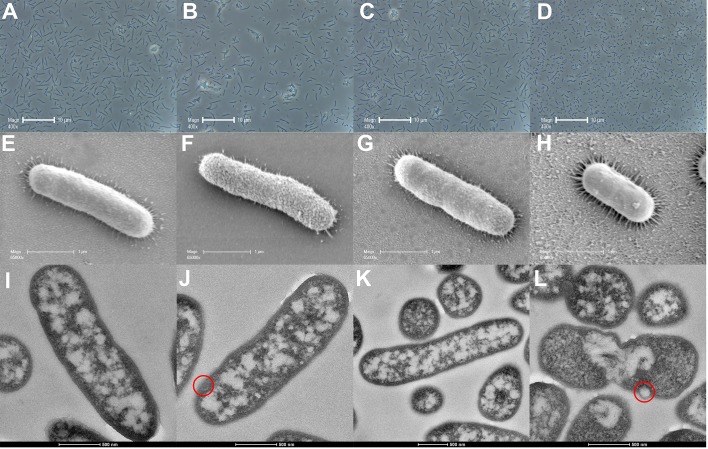
Images from phase contrast microscopy **(A–D)**, scanning electron microscopy **(E–H)**, and transmission electron microscopy **(I–L)** for polymyxin-susceptible *A. baumannii* ATCC 17978 treated with 2 mg/L polymyxin B **(B,F,J)**, 4 mg/L mitotane **(C,G,K)**, or both **(D,H,L)**. **A**, **E**, and **I** represent the control condition. Membrane blebs are indicated by red circles.

**Figure 2** shows phase contrast microscopy, SEM and TEM images of polymyxin-susceptible *A. baumannii* ATCC 17978 following treatment with polymyxin B (2 mg/L), mitotane (4 mg/L), or both. Phase contrast microscopy images showed that polymyxin B (**Figure 2B**) or mitotane (**Figure 2C**) monotherapy had minimal impacts on the overall morphology of the bacterial cells compared to the control group (**Figure 2A**); the average cell length remained approximately 2 μm in all cases. However, more clumps of cells were observed with polymyxin B monotherapy (**Figure 2B**). In combination (**Figure 2D**), polymyxin B and mitotane resulted in significantly shorter cells compared to the other groups with the average cell length reduced to approximately 1 μm. From SEM, polymyxin B monotherapy (**Figure 2F**) affected the integrity of the cell surface in polymyxin-susceptible *A. baumannii*. Without treatment (**Figure 2E**), the bacterial surface appeared even and smooth, while the surface became uneven and rough following treatment with polymyxin B (**Figure 2F**). Mitotane monotherapy (**Figure 2G**) and polymyxin B/mitotane combination therapy (**Figure 2H**) had minimal impacts on the bacterial surface, although the cell length was confirmed to be much shorter. TEM results reveal that polymyxin B monotherapy (**Figure 2J**) caused membrane blebbing. Compared to the control group (**Figure 2I**), treatment with mitotane monotherapy (**Figure 2K**) had little impact on the bacterial surface. Similar to polymyxin B monotherapy, membrane blebbing was also observed for the treatment with polymyxin B/mitotane combination (**Figure 2L**). Additionally, TEM images showed that bacterial cells treated with the polymyxin B/mitotane combination were much shorter in length and most appeared to be undergoing a cell division cycle, with evident chromosomal segregation.

**FIGURE 3:**
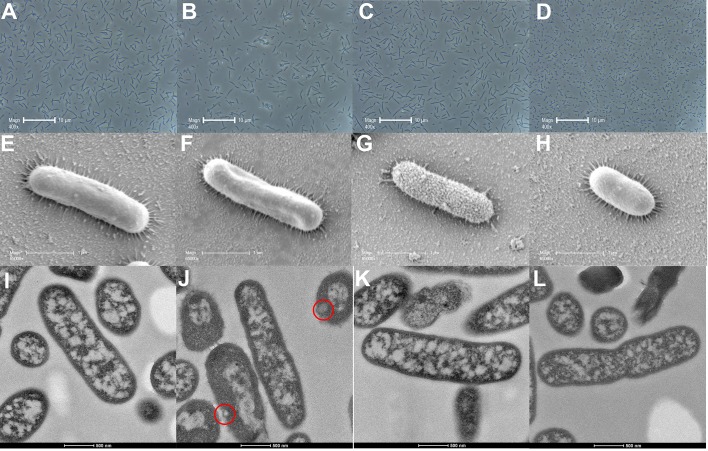
Images from phase contrast microscopy **(A–D)**, scanning electron microscopy **(E–H)**, and transmission electron microscopy **(I–L)** for polymyxin-resistant *A. baumannii* FADDI-AB225 treated with 2 mg/L polymyxin B **(B,F,J)**, 4 mg/L mitotane **(C,G,K)**, or both **(D,H,L)**. **A**, **E**, and **I** represent the control condition. Membrane blebs are indicated by red circles.

Phase contrast microscopy, SEM, and TEM images for polymyxin-resistant *A. baumannii* FADDI-AB225 treated with polymyxin B (2 mg/L), mitotane (4 mg/L), or both are shown in **Figure 3**. Similar to the results for polymyxin-susceptible *A. baumannii* ATCC 17978, phase contrast microscopy results showed no changes in bacterial size compared to the control group (**Figure 3A**) following treatment with polymyxin B (**Figure 3B**) and mitotane (**Figure 3C**) monotherapy, while the polymyxin B/mitotane combination (**Figure 3D**) led to a significant reduction in the cell length. For SEM, treatment with polymyxin B monotherapy (**Figure 3F**) did not affect the bacterial cell surface; however, the overall structure appeared distorted. Treatment with mitotane monotherapy (**Figure 3G**) affected the cell surface of polymyxin-resistant *A. baumannii* FADDI-AB225, as the surface was more uneven and rough compared to the control group (**Figure 3E**). Combination therapy (**Figure 3H**) did not affect the membrane surface, although it led to substantial shortening of the cells. For TEM, similar results to polymyxin-susceptible isolates were once again observed. Membrane blebbing was evident in bacteria treated only with polymyxin B (**Figure 3J**), but not in those treated only with mitotane (**Figure 3K**). With the polymyxin B/mitotane combination (**Figure 3L**), most cells were substantially shorter compared to the control group (**Figure 3I**) and appeared to be going through cell division. Unlike the polymyxin-susceptible isolate, no membrane blebbing was observed with the combination in the polymyxin-resistant isolate.

### *In Vivo* Antimicrobial Activity of Polymyxin B and Mitotane Against Polymyxin-Resistant *A. baumannii* FADDI-AB225 in a Mouse Burn Wound Infection Model

**FIGURE 4:**
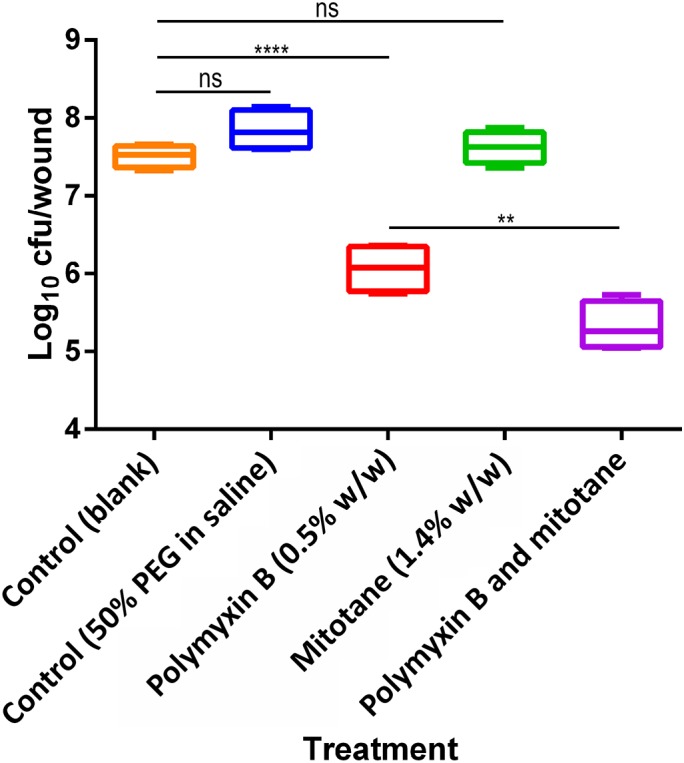
Efficacy of polymyxin B alone, mitotane alone, and the combination against polymyxin-resistant *A. baumannii* FADDI-AB225 in a mouse wound infection model. Statistical significance was calculated with one-way ANOVA and Tukey’s multiple comparisons (ns = *p* > 0.5, ^∗∗^ = *p* ≤ 0.01, and ^∗∗∗∗^ = *p* ≤ 0.0001). Box plots indicate upper and lower quartiles (top and bottom of box); median (line within box); and the spread of data (whiskers).

**Figure 4** shows the bacterial killing of polymyxin B (0.5%, w/w), mitotane (1.4%, w/w), and the polymyxin B/mitotane combination against polymyxin-resistant *A. baumannii* FADDI-AB225. One-way ANOVA showed significant difference between the means of all groups (*p* < 0.0001). There was no significant difference in the bacterial load between the blank control (i.e., no treatment) and solvent control groups (mean log_10_ cfu/wound difference, -0.33; Tukey’s HSD, *p* > 0.05), indicating that the solvent possessed no major antimicrobial activity. Although this isolate was polymyxin-resistant, topical polymyxin B (0.5%, w/w) monotherapy significantly reduced the bacterial load (mean log_10_ cfu/wound difference, -1.44 vs. blank control; Tukey’s HSD, *p* ≤ 0.0001). However, there was no significant reduction in the bacterial load (mean log_10_ cfu/wound difference, -0.11 vs. blank control; Tukey’s HSD, *p* > 0.5) with topical mitotane (1.4%, w/w) alone (**Figure 4**). Importantly, both agents used in combination produced a further significant reduction in the bacterial load compared to polymyxin B monotherapy (mean log_10_ cfu/wound difference, -0.74; Tukey’s HSD, *p* ≤ 0.01). Compared to the blank control group, the polymyxin B/mitotane combination resulted in a mean log_10_ cfu/wound difference of -2.19 (Tukey’s HSD, *p* ≤ 0.0001).

## Discussion

Given the rapid emergence of multidrug-resistance and the limited new effective antibiotics developed over the last two decades (Boucher et al., 2009, 2013), novel approaches for the treatment of MDR Gram-negative bacteria infections are urgently needed. This is the first study to investigate the potential utility of polymyxin B in combination with the FDA-approved antineoplastic mitotane to treat infections caused by polymyxin-resistant MDR Gram-negative pathogens. Mitotane is a derivative of the insecticide dichlorodiphenyl-trichloroethane and is currently used for the treatment of adrenocortical carcinoma (ACC) (Lalli, 2015). The precise mechanism of action of mitotane in ACC is not well understood, but it has been shown to inhibit the activity of sterol-*O*-acyl-transferase and induce endoplasmic reticulum (ER) stress in ACC cells (Sbiera et al., 2015). Our study is the first to demonstrate its potential application for the treatment of Gram-negative infections when combined with polymyxin B.

To ensure the applicability of the combination of polymyxin B and mitotane to a diverse population of problematic Gram-negative bacteria, three Gram-negative bacterial species (*A. baumannii*, *P. aeruginosa*, and *K. pneumoniae*) were selected for the initial *in vitro* antimicrobial activity evaluation. Isolates selected included MDR, carbapenem-resistant, and polymyxin-resistant strains with known different mechanisms of polymyxin resistance. *A. baumannii* and *P. aeruginosa* were selected as they are frequently resistant to multiple classes of antibiotics and are currently considered by the WHO as two of the top bacterial “superbugs” requiring urgent antibiotic development (Tacconelli and Magrini, 2017). *K. pneumoniae* was also examined as it is also identified as a top bacterial “superbug” by the WHO due to the rapid emergence of carbapenem resistance (including NDM production) (Yong et al., 2009; Kumarasamy et al., 2010; Farzana et al., 2013). Concentrations of 2 mg/L for polymyxin B and 4 m/L for mitotane were examined as they were achievable in patients (Hermsen et al., 2011; Sandri et al., 2013).

One of the major concerns surrounding the intravenous use of polymyxin B or colistin monotherapy for the treatment of infections caused by Gram-negative bacteria is the development of resistance *via* amplification of polymyxin-resistant subpopulations (Tam et al., 2005; Tan et al., 2007; Bergen et al., 2011; Meletis et al., 2011; Deris et al., 2012; Hermes et al., 2013; Lee et al., 2013; Ly et al., 2015; Lenhard et al., 2017; Zhao et al., 2017). Consequently, the use of antibiotic combination therapy represents a potential option to increase bacterial killing and prevent the emergence of polymyxin resistance as the combination may result in subpopulation or mechanistic synergy (Landersdorfer et al., 2013). Despite extensive bacterial killing by polymyxin B monotherapy against five polymyxin-susceptible isolates, regrowth with associated polymyxin resistance (the latter evident by significantly increased polymyxin B MICs compared to baseline values) subsequently occurred with three isolates (*A. baumannii* ATCC 19606, *A. baumannii* FADDI-AB180, and *K. pneumoniae* ATCC 13883; **Figure 1A**). When used as monotherapy, mitotane showed antimicrobial activity against only one isolate (**Figure 1B**). However, the combination of polymyxin B and mitotane significantly improved bacterial killing against the less susceptible isolates (i.e., those that were resistant to polymyxin B or mitotane monotherapy, or showed regrowth at 24 h; **Figures 1A,B**). The enhanced antimicrobial killing was indicated by the complete prevention of regrowth in all polymyxin-susceptible isolates after 24 h (**Figure 1A**) and >2 log_10_ cfu/mL reduction within the first 6-h treatment against the four polymyxin-resistant isolates compared to the more active monotherapy (**Figure 1B**). Although regrowth occurred in four of the five polymyxin-resistant isolates, the combination still enhanced initial bacterial killing which may assist with the bacterial clearance from the body. Since polymyxins are well known for their ability to permeabilize the outer membrane of Gram-negative bacteria (Salmelin et al., 2000; Tsubery et al., 2000; Sahalan and Dixon, 2008), a possible mechanism for the enhanced killing observed with the combination is permeabilization of the outer membrane by polymyxin B leading to the entry of mitotane into the bacterial cell. Indeed, polymyxin B and its derivative polymyxin B nonapeptide had previously been shown to enhance the antimicrobial activity of hydrophobic antibiotics against Gram-negative bacteria and yeasts (Ofek et al., 1994; Pietschmann et al., 2009). Interestingly, mitotane monotherapy displayed substantial antimicrobial activity against LPS-deficient, polymyxin-resistant *A. baumannii* FADDI-AB065 (**Figure 1B**). LPS in the outer membrane of Gram-negative bacteria acts as a highly selective permeability barrier that protects bacteria from harmful substances (Nikaido, 2003). Consequently, it is possible that in the absence of LPS, mitotane was able to enter bacterial cells and exert its antimicrobial activity. Another notable finding is that mitotane monotherapy also lowered the polymyxin B MIC of polymyxin-resistant *A. baumannii* FADDI-AB225 (**Table 2**); however, it did not affect the polymyxin B MICs of the other polymyxin-resistant isolates. The mechanism for this phenomenon is currently unclear, although it may result from the expression of LPS variants by the different isolates. Coincidently, it has been reported that *Moraxella catarrhalis* and *Salmonella typhimurium* with deep rough-type LPS displayed higher susceptibility to hydrophobic antimicrobial agents (Tsujimoto et al., 1999). Further mechanistic studies are warranted.

According to the SEM imaging results, it is possible that the polymyxin resistance in *A. baumannii* FADDI-AB225 altered their surface interaction with mitotane, as the outer membrane appeared disrupted (uneven and rough) following mitotane monotherapy in *A. baumannii* FADDI-AB225 (**Figure 3G**), but not *A. baumannii* ATCC 17978 (**Figure 2G**). Both the SEM and TEM images showed disruptive changes to the outer membrane of polymyxin-susceptible *A. baumannii* ATCC 17978 following polymyxin B monotherapy (**Figures 2F,J**), which confirmed the known impact of polymyxin B on the outer membrane of Gram-negative bacteria. For the lipid A modified polymyxin-resistant *A. baumannii* FADDI-AB225, no disruptive effect on the surface membrane by polymyxin B monotherapy was observed with SEM (**Figure 3F**), most likely due to the modification of lipid A which resulted in minimal polymyxin B affinity. Membrane blebs, however, were still observed by TEM in polymyxin-resistant *A. baumannii* FADDI-AB225 treated with 2 mg/L polymyxin B alone (**Figure 3J**), indicating that blebbing might not necessarily result in cell death, but was enough to allow the mitotane to enter and exert antibacterial effect. Although monotherapy with mitotane or polymyxin B appeared to impact the outer membrane of polymyxin-resistant and -susceptible *A. baumannii*, the combination impacted the overall structure of both strains leading to an extensive shortening in the length of the bacteria (**Figures 2, 3**). SEM images showed a smooth membrane surface on the shortened bacterial cells, suggesting that the combination prevented the formation of the rough surface, which could be an adaptive response to polymyxin B or mitotane monotherapy. Numerous incompletely separated cells revealed by TEM images (**Figures 2L, 3L**) suggest a possible impact on the bacterial DNA replication.

In our mouse burn wound infection study, the combination displayed effective antimicrobial activity against polymyxin-resistant *A. baumannii*. The doses of 5 mg/kg for polymyxin B (subcutaneous median lethal dose in mice [LD_50_] 59 mg/kg) and 14 mg/kg for mitotane (oral LD_50_ > 4,000 mg/kg in mice, dermal LD_50_ not available) were selected, as they are safe in animals according to their material safety data sheets. Based on the available LD_50_ limits of polymyxin B and mitotane, it is likely that much higher doses of both drugs can be used for topical combination therapy. Given the lack of an optimized topical formulation, it is possible that the *in vivo* efficacy of the combination in the current study was underestimated. Nevertheless, the combination treatment was able to significantly reduce the number of polymyxin-resistant *A. baumannii*, compared to polymyxin B or mitotane monotherapy.

## Conclusion

Our study is the first to reveal the synergistic activity of mitotane, an FDA-approved non-antibiotic drug, in combination with polymyxin B against problematic Gram-negative bacteria. Importantly, the combination also prevented the emergence of polymyxin resistance. As mitotane is currently used in humans, its repositioning for antimicrobial purposes may be easier than discovering novel antibacterial compounds against Gram-negative “superbugs”. The synergistic antibacterial killing of polymyxin B with mitotane in animals raises hopes for the potential repositioning of mitotane against MDR Gram-negative bacteria and further clinical investigations are warranted.

## Ethics Statement

This study was carried out in accordance with the recommendations of “Australian Code of Practice for the Care and Use of Animals for Scientific Purposes” and Monash Institute of Pharmaceutical Sciences Animal Ethics Committee. The protocol was approved by the Monash Institute of Pharmaceutical Sciences Animal Ethics Committee before the study started.

## Author Contributions

TT carried out the main experiments, data analysis, and wrote the manuscript draft. JW participated in the animal study. PB participated in *in vitro* studies’ design. YD and TV participated in data analysis. JL designed the project and guided all experimental designs and data analysis. All authors participated in manuscript revision and read and approved the final manuscript.

## Conflict of Interest Statement

The content is solely the responsibility of the authors and does not necessarily represent the official views of the National Institute of Allergy and Infectious Diseases or the National Institutes of Health. JL is an Australian National Health and Medical Research Council (NHMRC) Senior Research Fellow. TV is an Australian NHMRC Industry Career Development Research Fellow. The other authors declare that the research was conducted in the absence of any commercial or financial relationships that could be construed as a potential conflict of interest.
